# Risk Factor Analysis of Complications and Mortality Following Coil Procedures in Patients with Intracranial Unruptured Aneurysms Using a Nationwide Health Insurance Database

**DOI:** 10.3390/jcm13041094

**Published:** 2024-02-15

**Authors:** So Yeon Park, So An Kim, Yu Hyeon An, Sang Won Kim, Saeyoon Kim, Jae Min Lee, Youngjin Jung

**Affiliations:** 1 Department of Medicine, College of Medicine, Yeungnam University, Daegu 42415, Republic of Korea; 2 Medical Research Center, College of Medicine, Yeungnam University, Daegu 42415, Republic of Korea; 3 Department of Pediatrics, College of Medicine, Yeungnam University, Daegu 42415, Republic of Korea; sysnow88@ynu.ac.kr; 4 Department of Neurosurgery, Yeungnam University Medical Center, Daegu 42415, Republic of Korea

**Keywords:** unruptured intracranial hemorrhage, Charlson Comorbidity Index, intracranial hemorrhage, intracranial infarction

## Abstract

(1) Background: Unruptured intracranial aneurysm (UIA) occurs in 1–2% of the population and is being increasingly detected. Patients with UIA are treated with close observation, endovascular coiling or surgical clipping. The proportion of endovascular coiling has been rising. However, complications such as cerebral infarction (CI), intracranial hemorrhage (ICRH), and death remain crucial issues after coil treatment. (2) Methods: We analyzed the incidence and risk factors of complications after the use of coil in patients with UIA based on the patients’ characteristics. We utilized the Health Insurance Review and Assessment (HIRA) database. Patients treated with coils for UIA between 1 January 2015 and 1 December 2021 were retrospectively analyzed. (3) Results: Of the total 35,140 patients, 1062 developed ICRH, of whom 87 died, with a mortality rate of 8.2%. Meanwhile, 749 patients developed CI, of whom 29 died, with a mortality rate of 3.9%. The overall mortality rate was 1.8%. In a univariate analysis of the risk factors, older age, males, a higher Charlson Comorbidity Index (CCI) score, and diabetes increase the risk of CI. Meanwhile, males with higher CCI scores and hemiplegia or paraplegia show increased ICRH risk. Older age, males and metastatic solid tumors relate to increased mortality risk. (4) Conclusions: This study is significant in that the complications based on the patient’s underlying medical condition were analyzed.

## 1. Introduction

### 1.1. Unruptured Intracranial Aneurysm

Unruptured intracranial aneurysm (UIA) is an acquired lesion that occurs in 1–2% of the population and accounts for 80% of non-traumatic subarachnoid hemorrhage causes [1]. UIA is increasingly detected with the growing use of imaging techniques such as computerized tomography (CT) and magnetic resonance imaging (MRI). Moreover, it is usually detected at the internal carotid, anterior communicating, anterior cerebral, and middle cerebral arteries. Twenty percent of patients have more than one aneurysm [2]. A few factors increase the occurrence of aneurysms. Untreatable risk factors include old age, female sex, and genetic factors, while treatable factors include smoking and hypertension. Genetic conditions, such as autosomal dominant polycystic kidney disease, increase the incidence of UIA [3]. The overall prevalence increased by up to 10% when other family members had brain aneurysms.

A potential aneurysm can be detected through CT or MRI, and further clarification of its location and morphological data requires Magnetic Resonance Angiography (MRA) or Computed Tomography Angiography (CTA). UIA may remain asymptomatic for several years. However, as the UIA enlarges, it can exert pressure on adjacent central nervous system structures, leading to hemiparesis, seizures, cranial nerve palsy, and, in rare cases, an embolus in the aneurysmal sac that can cause cerebral infarction (CI) [4]. Moreover, UIA has the potential to cause aneurysmal subarachnoid hemorrhage, which has a mortality rate of 35% in Europe [5].

### 1.2. Endovascular Coiling

Patients with UIA are often treated with endovascular coiling or surgical clipping [6]. Upon the detection of a UIA, several factors should be considered to establish the optimal approach for management. The risk of aneurysmal rupture without any intervention should be compared with the risk of endovascular coiling or surgical clipping. The selection of intervention is based on aneurysmal factors such as aneurysm size, location, and the nature of the neck of the aneurysm, as well as patient factors such as age, history of subarachnoid hemorrhage, and family history [2]. Endovascular coiling is frequently preferred over clipping due to its lower risk [7,8,9]. Despite advances in endovascular coiling due to developments in microcatheter technology, there remain considerable complications, such as CI and intracranial hemorrhage (ICRH) [10].

### 1.3. Aim

Previous studies have analyzed complications and mortality after treatment in patients with UIA; however, only a few studies have analyzed the mortality after complications in patients with UIA who underwent coil treatment or compared the risk of complications and mortality based on patient risk factors. Therefore, this retrospective cohort study aimed to analyze the incidence of complications and mortality after coil treatment in patients with UIA according to patient characteristics using the Health Insurance Review and Assessment (HIRA) database. The comparative analysis of the association between specific risk factors and ICRH, CI, and mortality contributes to reducing the risk of complications and aids in determining optimal treatment for patients with UIA.

## 2. Materials and Methods

### 2.1. Unruptured Intracranial Aneurysm

This study utilized a patient sample sourced from the HIRA database. HIRA is a national medical evaluation organization established to accurately review medical expenses, evaluate the adequacy of medical benefits for the National Health Insurance, and provide data on medical treatment information, medicines, and medical resources nationwide. The data requested from HIRA includes patient medical history, treatment, drug usage, and surgery history, and are utilized for evidence-based national healthcare policy development and academic research [11,12,13]. Events such as hemorrhage, infarction, and death included all those that occurred during the same period of hospitalization for the coil procedure.

### 2.2. Study Population 

**Figure 1:**
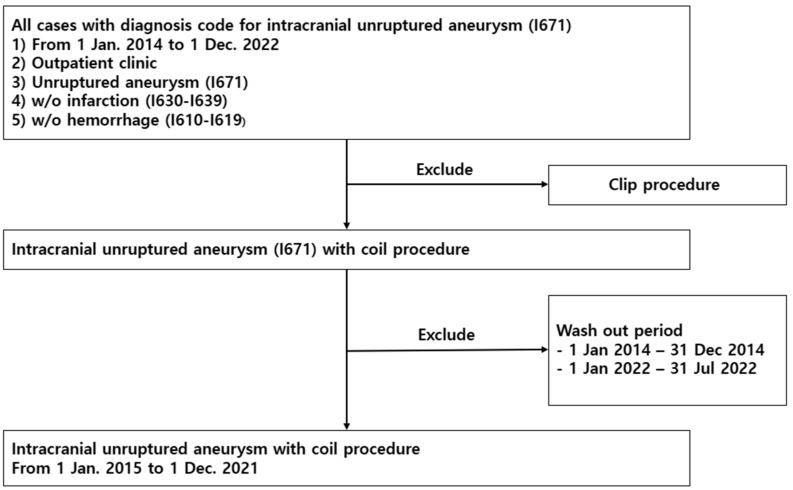
Flowchart for patient selection.

The diagnosis was based on the 7th Korean Classification (KCD-7), a revision of the International Classification of Diseases (ICD-10), and UIA was coded as I671. To extract information on patients with UIA, newly diagnosed patients of all ages between 1 January 2014 and 31 July 2022 were included. We examined patients who visited the outpatient clinic without infarction codes (I630–639) or hemorrhage codes (I610–I619). Subsequently, we excluded patients who underwent clip procedures and washed out the period from 1 January 2014 to 31 December 2014, and from 1 January 2022 to 31 July 2022. Finally, patients who had UIA with a coil procedure between 1 January 2015 and 1 December 2021 were analyzed (Figure 1). Charlson’s comorbidities index (CCI) score was calculated based on the ICD-10 diagnosis codes of UIA patients [14].

### 2.3. Statistical Analyses

Data are represented as the mean ± standard deviation. Multiple logistic regression analysis was used to analyze the risk factors for hemorrhage, infarction, and death. *p* values < 0.05 were considered statistically significant. Statistical analysis was performed using SAS enterprise guide version 9.4 (SAS Cary, NC, USA). The annual incidence rate is per 100,000 population, directly age-adjusted to the 2021 population.

## 3. Results

### 3.1. Patient Characteristics

During the study period, 35,140 patients were treated with coils for UIA. Of these, 3304 (9.4%) were aged 44 years or younger, 19,047 (54.2%) were aged 45–64 years, 9001 (25.6%) were aged 65–74 years, and 3788 (10.7%) were aged >75 years (Table 1). The mean age of the patients was 60.6 years. Females constituted 25,411 (72.3%) of patients, while males comprised 9729 (27.7%).

The most common underlying disease among patients was peptic ulcer disease (18.7%), followed by mild liver disease (11.9%), and diabetics without chronic complications (11.4%). The remaining underlying diseases include chronic pulmonary disease (11.0%), peripheral vascular disease (5.9%), any malignancy including leukemia and lymphoma (4.7%), congestive heart failure (3.8%), hemiplegia or paraplegia (2.7%), renal disease (2.3%), dementia (1.5%), rheumatologic disease (1.5%), metastatic solid tumor (0.9%), myocardial infarction (0.8%), moderate or severe liver disease (0.1%), and diabetes with chronic complications (0.0%).

### 3.2. Complications and Mortality after the Procedure

Of the 35,140 patients who underwent coil procedures, ICRH occurred in 1062 patients, 87 of whom died, with a mortality rate of 8.2% (Table 2). Meanwhile, of the 749 patients who developed a CI, 29 died, representing a 3.9% mortality rate. Of the 33,329 patients without complications of ICRH or CI, 519 died, with a mortality rate of 1.6%. The total number of deaths was 635, representing a 1.8% mortality rate.

The length of hospitalization was 26.4 ± 18.2 days: 26.4 ± 16.4 and 26.4 ± 18.3 days for patients with and without complications, respectively.

### 3.3. Risk Factor for Infarction of an Unruptured Intracranial Aneurysm

In univariate analysis, the older age group, male sex (OR = 1.778), higher CCI score, myocardial infarction (OR = 3.157), congestive heart failure (OR = 2.385), dementia (OR = 2.496), chronic pulmonary disease (OR = 1.392), diabetes without chronic complication (OR = 1.6), diabetes with chronic complication (OR = 44.882), hemiplegia or paraplegia (OR = 6.976), renal disease (OR = 2.208), any malignancy including leukemia and lymphoma (OR = 1.562), and metastatic solid tumor (OR = 2.020) were associated with a significant increase in the risk for CI (Table 3). In multivariate analysis, the older age group, male sex (OR = 1.827), myocardial infarction (OR = 2.084), diabetes without chronic complication (OR = 1.272), diabetes with chronic complication (OR = 18.814), and hemiplegia or paraplegia (OR = 3.933) were significantly associated with increased risk for CI.

### 3.4. Risk Factor of Hemorrhage from an Unruptured Intracranial Aneurysm

In univariate analysis, males sex (OR = 1.396), the higher CCI group, diabetes without chronic complications (OR = 1.211), hemiplegia or paraplegia (OR = 11.986), and renal disease (OR = 1.444) were significantly associated with increased risk for ICRH. Meanwhile, in multivariate analysis, male sex (OR = 1.331) and those with hemiplegia or paraplegia (OR = 7.888) were significantly associated with an increased risk for ICRH (Table 4).

### 3.5. Risk Factor of Death by Intracranial Unruptured Aneurysm after Coil Procedure

In the univariate analysis, the older age group, male sex (OR = 1.526), myocardial infarction (OR = 3.278), congestive heart failure (OR = 2.919), dementia (OR = 2.587), chronic pulmonary disease (OR = 1.783), mild liver disease (OR = 1.275), diabetes without chronic complications (OR = 1.307), hemiplegia or paraplegia (OR = 3.178), renal disease (OR = 2.601), any malignancy including leukemia and lymphoma (OR = 7.073), moderate or severe liver disease (OR = 8.616), and metastatic solid tumor (OR = 20.814) were significantly associated with increased death risk. Meanwhile, in multivariate analysis, older age group, male sex (OR = 1.510), myocardial infarction (OR = 2.058), hemiplegia or paraplegia (OR = 1.968), any malignancy including leukemia and lymphoma (OR = 2.760), moderate or severe liver disease (OR = 5.030), and metastatic solid tumor (OR = 4.651) were significantly associated with increased death risk (Table 5).

## 4. Discussions

In this study, 35,140 patients with UIA with a mean age of 60.6 years, treated with coil between 2015 and 2021, were enrolled. Females comprise 25,411 (72.3%), and 19,047 (54.2%) patients were aged 45–64 years.

In a European population-based prevalence study, 2000 individuals were screened using brain MRI, and 1.8% of adult participants were observed to have an intracranial aneurysm [15]. In a study based on a United States (US) nationwide inpatient sample from 1998 to 2003, the average age of patients treated with coil for UIA was 56.8 years. The proportion of female patients undergoing coil treatment for UIA was 75.8% [16]. Naggara et al. conducted a systematic review and meta-analysis of coil procedures for UIA between January 2003 and July 2008 and reported that 78% of patients with UIA were female, with an average age of 52.3 years [17]. In a study of patients with UIA in the US, Canada, and Europe, Wiebers et al. reported that 351 (77.8%) of 451 patients treated with coil were women, with a mean age of 53.7 years [18]. In a Korean multicenter retrospective study of 2180 patients with UIA between 2007 and 2009, the patients had a mean age of 58.6 years, and 70.5% were female [19].

In our study, the rate of ICRH in patients who underwent coil was 3.0%, and the rate of CI was 2.1%. The overall mortality was 1.8%, and the mortality in patients with complications was 6.4%

Previous research has indicated that among older adult patients with UIA (aged >65 years) who underwent coil treatment, the incidence of ICRH was 0.3–3.9% and the incidence of CI was 2.9–8.9% [20,21,22,23,24,25]. Moreover, the mortality rate within 30 days was 0–1.9%, and within a year of treatment, it was 7.6–10% [20,21,22,24,25,26,27]. McDonald et al. analyzed the efficacy of 4899 UIA in the US using a healthcare database and reported a 2.0% rate of ICRH, a 3.6% rate of ischemic complications, and a 0.5% rate of in-hospital mortality in patients who underwent coil treatment [28].

In a Korean nationwide cohort study, the incidence of hemorrhagic complications, such as intraoperative rupture, had been reported to range from 0.0% to 9.5%, following coil treatment for UIA [29]. The incidence of ischemic complications, such as thromboembolic events, or CI, ranges from 1.08% to 16.6%. Furthermore, the incidence of neurologic impairments resulting from procedure-related complications, such as infarction or hemorrhagic complications, ranges from 0.27 to 14.7%, and the death rate is estimated to be between 0.0 and 1.4%.

In a study by Lee et al. using the Korean National Health Insurance Service database, the frequency of ICRH requiring secondary surgery in patients with UIA receiving coil treatment between 2013 and 2016 was 0.99% [30]. In the same study, 10.1% of patients who underwent coil treatment developed a CI. Patients with perioperative ICRH or CI who died within a year of treatment comprised 1.16% of the coil group. The rate for ICRH is higher in our study than that reported by Lee et al., which may be attributed to the fact that they defined hemorrhage as requiring additional surgery, such as hematoma drainage, craniotomy, or craniectomy, to exclude minor bleeding.

In a study by Chang et al. analyzing patients with UIA from 2010 to 2014 utilizing the Korean HIRA database, 13,756 patients were treated with the coil, with an average hospital stay of 8.6 ± 7.4 days [31]. Familiari et al. investigated the duration of hospital stay after coil application in patients with UIA with a diameter of ≥25 mm, and observed a mean length of intensive care unit stay of 7 days and a mean length of general ward stay of 10.5 days [32]. In our study, the total mean length of hospitalization was 26.4 ± 18.2 days, with 26.4 ± 16.4 days for patients with complications.

Several studies have been conducted regarding risk factors for CI, ICRH, or death after coil treatment, such as the size of the aneurysm and the age of patients. The larger the aneurysm size, the higher the risk of CI and ICRH [33,34,35]. However, there is a lack of research on the relationship between underlying diseases and CI/ICRH.

CCI is the most widely used tool to predict the mortality of a patient based on their comorbidities, such as myocardial infarction, congestive heart failure, or cancer [36,37,38,39]. Although Lee et al. reported that patients with a high CCI had a lower rupture rate of UIA [40], there are few studies on the correlation between CCI scores and complication rates after coil procedures in patients with UIA.

Our study holds significance in its analysis of the impact of the CCI scores of patients with UIA on the incidence of complications after coil treatment. Moreover, we observed that older age, male sex, a higher CCI score, myocardial infarction, congestive heart failure, dementia, chronic pulmonary disease, diabetes, hemiplegia or paraplegia, renal disease, malignancy, and metastatic solid tumors were significantly associated with increased risk for CI after coil treatment. Meanwhile, males in the higher CCI group with diabetes, hemiplegia or paraplegia, and renal disease were associated with increased ICRH risk after coil treatment. Our study observed that older age, male sex, myocardial infarction, congestive heart failure, dementia, chronic pulmonary disease, mild liver disease, diabetes, hemiplegia or paraplegia, renal disease, malignancy, and liver disease were associated with increased mortality after coil treatment.

In our study, the length of hospitalization was 26.4 ± 16.4 and 26.4 ± 18.3 days for patients with and without complications, respectively. Due to the nature of the HIRA database, we could not confirm the amount of hemorrhage or the extent of infarction, so it is possible that many cases with relatively mild hemorrhage and infarction were included. Further research is needed in this area.

Older adults aged >65 years and female sex [41], age over 40 years [42], male and diabetes [43], and autosomal dominant polycystic kidney disease [44] are associated with increased complications such as CI and ICRH after coil treatment in patients with UIA. Wiebers et al. evaluated patients who visited the ISUIA center in the USA, Canada, and Europe. Among 451 patients who were treated with coil for UIA, patients aged >50 years or those with posterior circulation aneurysms are more likely to have poor endovascular outcomes 1 year after coil treatment [18]. In total, 8 (2.0%) surgery-related deaths at 30 days and 14 (3.4%) deaths at 1 year after treatment were reported. Mahaney et al. studied 451 patients enrolled in the International Study of Unruptured Intracranial Aneurysms for UIA; they reported that a one-year risk of death after coil treatment increases with age [21].

Our study has certain limitations. First, due to the nature of the HIRA database, we did not have information on the location and size of the aneurysm, and we were unable to verify the patient’s clinical information in detail. Second, this study may have a selection bias because of its retrospective design. Third, patients who died after discharge were not included. Fourth, there is no comparison of outcomes with untreated UIA.

## 5. Conclusions

In our study, we analyzed the characteristics of patients who underwent coil for UIA and identified the risk factors of ICRH, CI, and death after coil procedures. Few studies have analyzed the impacts of underlying medical conditions on complications and mortality following coil procedures. Our study is unique in that we analyzed the complications based on the patient’s underlying medical condition, regardless of the size or location of the UIA. In the next study, we plan to investigate the treatment results of applying a clip in UIA, and compare and analyze the differences in treatment results between clip and coil procedures in UIA.

## Figures and Tables

**Table 1:** Characteristics of patients.

Variable		N (%)
Total number of patients		35,140 (100)
Age (years)		60.6 ± 0.5
Age group (years)		
	≤44	3304 (9.4)
	45–64	19,047 (54.2)
	65–74	9001 (25.6)
	≥75	3788 (10.7)
Sex		
	Male	9729 (27.7)
	Female	25,411 (72.3)
Underlying disease		
	Myocardial infarction	286 (0.8)
	Congestive heart failure	1340 (3.8)
	Peripheral vascular disease	2084 (5.9)
	Dementia	540 (1.5)
	Chronic pulmonary disease	3860 (11.0)
	Rheumatologic disease	530 (1.5)
	Peptic ulcer disease	6580 (18.7)
	Mild liver disease	4187 (11.9)
	Diabetes without chronic complication	4007 (11.4)
	Diabetes with chronic complication	2 (0.0)
	Hemiplegia or paraplegia	954 (2.7)
	Renal disease	792 (2.3)
	Any malignancy including leukemia and lymphoma	1649 (4.7)
	Moderate or severe liver disease	22 (0.1)
	Metastatic solid tumor	313 (0.9)
Complication		
	Infarction	1062 (3.0)
	Hemorrhage	749 (2.1)
	No complication	33,329 (94.8)
Charlson comorbidity index		
	0	24,070 (68.5)
	1	3174 (9.0)
	2	5501 (15.7)
	3	1153 (3.3)
	≥4	1242 (3.5)

Patients with a CCI score of 0 numbered 20,470 (68.5%), those with 1 numbered 3174 (9.0%), those with 2 numbered 5501 (15.7%), those with 3 numbered 1153 (3.3%), and those with ≥4 numbered 1242 (3.5%).

**Table 2:** Complications and mortality after coil procedure in patients with intracranial unruptured aneurysm.

		Number of Patients, N	Length of Hospitalization (Days)	Mortality, N (%)
Total		35,140	26.4 ± 18.2	635 (1.8)
	Complication	1811	26.4 ± 16.4	116 (6.4)
	Hemorrhage	1062	26.7 ± 16.5	87 (8.2)
	Infarction	749	26.4 ± 16.3	29 (3.9)
	No complication	33,329	26.4 ± 18.3	519 (1.6)

**Table 3:** Risk factor of infarction of intracranial unruptured aneurysm.

				Univariate Analysis	Multivariate Analysis
		N	Annual Incidence Rate *	OR (95% CI)	OR (95% CI)
Age group (age)					
	<44 (ref)	31	0.12		
	45–64	311	1.84	1.757 (1.212–2.546)	1.715 (1.181–2.490)
	65–74	253	4.92	3.063 (2.105–4.457)	2.786 (1.906–4.072)
	≥75	154	4.15	4.502 (3.052–6.641)	3.846 (2.587–5.717)
Sex					
	Female (ref)	450	1.75		
	Male	29	0.11	1.778 (1.533–2.062)	1.827 (1.568–2.128)
Charlson comorbidity index					
	0 (ref)	598	2.31		
	1	89	0.34	1.551 (1.220–1.973)	1.803 (1.120–2.887)
	2	262	1.01	1.859 (1.548–2.233)	1.674 (0.912–3.073)
	3	43	0.17	2.290 (1.662–3.154)	2.443 (0.991–6.020)
	≥4	70	0.27	2.640 (1.966–3.545)	1.919 (0.518–7.102)
Underlying disease					
	None (ref)				
	Myocardial infarction	18	0.03	3.157 (1.947–5.119)	2.084 (1.262–3.440)
	Congestive heart failure	63	0.12	2.385 (1.831–3.106)	1.255 (0.663–2.372)
	Peripheral vascular disease	54	0.10	1.237 (0.934–1.638)	1.099 (0.826–1.463)
	Dementia	27	0.05	2.496 (1.683–3.703)	1.230 (0.611–2.479)
	Chronic pulmonary disease	109	0.21	1.392 (1.133–1.711)	0.755 (0.489–1.164)
	Rheumatologic disease	8	0.02	0.710 (0.352–1.432)	0.487 (0.222–1.068)
	Peptic ulcer disease	152	0.29	1.094 (0.913–1.310)	1.031 (0.858–1.239)
	Mild liver disease	64	0.12	0.684 (0.528–0.885)	0.432 (0.230–0.813)
	Diabetes without chronic complication	126	0.24	1.600 (1.317–1.944)	1.272 (1.041–1.553)
	Diabetes with chronic complication	1	0.00	44.882 (2.805–718.247)	18.814 (1.097–322.786)
	Hemiplegia or paraplegia	92	0.18	6.976 (5.535–8.793)	3.933 (2.123–7.286)
	Renal disease	35	0.07	2.208 (1.560–3.124)	1.016 (0.630–1.637)
	Any malignancy including leukemia and lymphoma	53	0.10	1.562 (1.176–2.075)	0.846 (0.452–1.583)
	Moderate or severe liver disease	1	0.00	2.123 (0.285–15.786)	2.598 (0.315–21.416)
	Metastatic solid tumor	13	0.03	2.020 (1.154–3.538)	1.187 (0.500–2.819)

* All rates are per 100,000 population, directly age-adjusted to the 2021 population.

**Table 4:** Risk factor of hemorrhage of intracranial unruptured aneurysm.

				Univariate Analysis	Multivariate Analysis
		N	Annual Incidence Rate *	OR (95% CI)	OR (95% CI)
Age group					
	<44 (ref)	91	0.35		
	45–64	574	3.39	1.105 (0.883–1.383)	1.123 (0.893–1.411)
	65–74	274	5.33	1.131 (0.889–1.438)	1.095 (0.855–1.403)
	≥75	123	3.31	1.225 (0.930–1.613)	1.099 (0.826–1.464)
Sex					
	Female (ref)	364	1.41		
	Male	698	2.70	1.396 (1.227–1.588)	1.331 (1.164–1.522)
Charlson comorbidity index					
	0 (ref)	598	1.16		
	1	89	0.17	1.143 (0.912–1.433)	1.230 (0.809–1.868)
	2	262	0.51	1.992 (1.717–2.311)	1.598 (0.888–2.875)
	3	43	0.08	1.554 (1.134–2.131)	1.544 (0.639–3.732)
	≥4	70	0.14	2.412 (1.869–3.111)	2.163 (0.615–7.601)
Underlying disease					
	None (ref)				
	Myocardial infarction	10	0.02	1.219 (0.646–2.299)	1.164 (0.605–2.239)
	Congestive heart failure	41	0.08	1.043 (0.760–1.433)	0.668 (0.351–1.270)
	Peripheral vascular disease	60	0.12	0.953 (0.732–1.242)	0.954 (0.728–1.250)
	Dementia	21	0.04	1.348 (0.867–2.094)	0.775 (0.381–1.578)
	Chronic pulmonary disease	118	0.23	1.022 (0.841–1.241)	0.849 (0.579–1.247)
	Rheumatologic disease	22	0.04	1.391 (0.904–2.140)	1.307 (0.766–2.230)
	Peptic ulcer disease	134	0.26	0.620 (0.516–0.745)	0.609 (0.505–0.734)
	Mild liver disease	115	0.22	0.888 (0.730–1.081)	0.596 (0.326–1.092)
	Diabetes without chronic complication	141	0.27	1.211 (1.011–1.451)	1.081 (0.896–1.305)
	Diabetes with chronic complication	0	0.00	0 (0–999)	0 (0–999)
	Hemiplegia or paraplegia	206	0.40	11.986 (10.106–14.216)	7.888 (4.376–14.219)
	Renal disease	33	0.06	1.444 (1.013–2.058)	1.234 (0.781–1.952)
	Any malignancy including leukemia and lymphoma	47	0.09	0.950 (0.706–1.278)	0.636 (0.343–1.178)
	Moderate or severe liver disease	0	0.00	0 (0–999)	0 (0–999)
	Metastatic solid tumor	11	0.02	1.197 (0.653–2.192)	0.888 (0.372–2.122)

* All rates are per 100,000 population, directly age-adjusted to the 2021 population.

**Table 5:** Risk factor of death of intracranial unruptured aneurysm.

				Univariate Analysis	Multivariate Analysis
		N	Annual Incidence Rate *	OR (95% CI)	OR (95% CI)
Age group					
	<44 (ref)	15	0.06		
	45–64	197	1.16	2.291 (1.354–3.878)	2.066 (1.217–3.506)
	65–74	215	4.18	5.365 (3.174–9.069)	4.474 (2.634–7.598)
	≥75	208	5.60	12.744 (7.530–21.566)	10.372 (6.088–17.673)
Sex					
	Female (ref)	233	0.90		
	Male	402	1.55	1.526 (1.297–1.797)	1.510 (1.269–1.796)
Charlson comorbidity index					
	0 (ref)	274	0.53		
	1	54	0.10	1.503 (1.120–2.017)	1.294 (0.855–1.958)
	2	129	0.25	2.085 (1.688–2.576)	1.329 (0.878–2.010)
	3	39	0.08	3.040 (2.162–4.275)	1.611 (0.862–3.011)
	≥4	139	0.27	10.944 (8.846–13.54)	1.989 (0.869–4.552)
Underlying disease					
	None (ref)				
	Myocardial infarction	16	0.03	3.278 (1.967–5.461)	2.058 (1.207–3.511)
	Congestive heart failure	64	0.12	2.919 (2.241–3.802)	1.430 (0.920–2.222)
	Peripheral vascular disease	36	0.07	0.952 (0.678–1.337)	0.813 (0.574–1.154)
	Dementia	24	0.05	2.587 (1.705–3.926)	1.147 (0.662–1.987)
	Chronic pulmonary disease	111	0.21	1.738 (1.412–2.139)	1.009 (0.725–1.404)
	Rheumatologic disease	13	0.03	1.376 (0.789–2.398)	1.182 (0.641–2.179)
	Peptic ulcer disease	114	0.22	0.949 (0.773–1.164)	0.766 (0.618–0.950)
	Mild liver disease	93	0.18	1.275 (1.021–1.592)	0.912 (0.605–1.373)
	Diabetes without chronic complication	91	0.18	1.307 (1.044–1.636)	0.929 (0.734–1.176)
	Diabetes with chronic complication	0	0.00	0 (0–999)	0 (0–999)
	Hemiplegia or paraplegia	50	0.10	3.178 (2.363–4.273)	1.968 (1.247–3.106)
	Renal disease	35	0.07	2.601 (1.836–3.684)	1.117 (0.735–1.698)
	Any malignancy including leukemia and lymphoma	154	0.30	7.073 (5.856–8.541)	2.760 (1.820–4.185)
	Moderate or severe liver disease	3	0.01	8.616 (2.543–29.189)	5.030 (1.312–19.284)
	Metastatic solid tumor	79	0.15	20.814 (15.914–27.223)	4.651 (2.797–7.736)

* All rates are per 100,000 population, directly age-adjusted to the 2021 population.

## Data Availability

The data presented in this study are available on request from the corresponding author.
